# Hyperphenylalaninemias genotyping: Results of over 60 years of history in Lombardy, Italy

**DOI:** 10.1002/edm2.396

**Published:** 2022-12-19

**Authors:** Valentina Rovelli, Graziella Cefalo, Vittoria Ercoli, Juri Zuvadelli, Turri Olivia, Daniela Graziani, Alberti Luisella, Davide Bassi, Alice Re Dionigi, Raed Selmi, Sabrina Paci, Elisabetta Salvatici, Giuseppe Banderali

**Affiliations:** ^1^ Clinical Department of Pediatrics, Inborn Errors of Metabolism Unit, San Paolo Hospital, ASST Santi Paolo e Carlo University of Milan Milan Italy; ^2^ Department of Laboratory Diagnostic Technologies San Paolo Hospital, ASST Santi Paolo e Carlo Milan Italy; ^3^ Regional Laboratory of Newborn Screening, Department of Women, Mothers and Neonatal Care, Children's Hospital “V. Buzzi” ASST Fatebenefratelli Sacco Milan Italy

**Keywords:** genotype, hyperphenylalaninemia, PAH, phenotype, phenylketonuria, PKU

## Abstract

**Background:**

Hyperphenylalaninemias (HPA) are due to several gene mutations, of which the *PAH* gene is the most frequently involved. Prevalence and incidence of disease vary between populations, with genotype/phenotype correlations not always capable to correctly predict disease severity. The aim of this study was to give an overview of *PAH* mutations among one of the largest cohort of patients among Europe, born in Lombardy (Italy) starting from late 1970 s and including over a 60 years of activity; furthermore, to evaluate and discuss identified genotype/phenotype correlations and related reliability.

**Patients/Methods:**

Eight hundred and twenty‐six HPA patients in current follow‐up at the San Paolo Hospital in Milan (Italy) were retrospectively reviewed, including molecular results and allelic phenotype and genotype values (attributed on the basis of the APV/GPV system) to verify genotype–phenotype correlations.

**Results:**

A total of 166 different *PAH* variants were reviewed; of those, seven variants were identified as not previously described in literature. Most frequently reported variant was p.Ala403Val, followed by p.Arg261Gln, p.Val245Ala, IVS10‐11 g>a, p.Tyr414Cys and p.Leu48Ser. Phenotype prediction, based on APV/GPV, matched the actual phenotype in most cases, but not always.

**Conclusion/Discussion:**

The cohort of patients included in this study constitute a representative sample of the HPA population worldwide. Studies on this sample may allow to improve clinical and genetic evaluation performances for affected patients, consequently to develop personalized medicine interventions and provide more precise indications on the correct treatment approach based on the accumulated evidence, also in light of a prognostically reliable but not always conclusive APV/GPV system.

## Graphical abstract

An overview of PAH mutations among one of the largest cohort of patients among Europe to evaluate and discuss identified genotype/phenotype correlations and related reliability. APV/GPV system is a prognostically reliable but not always conclusive system.

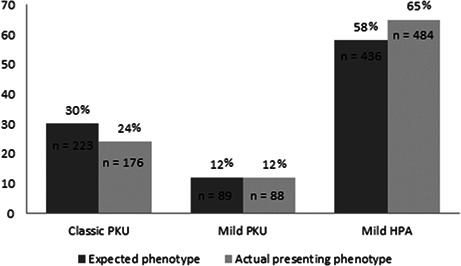

## 1 INTRODUCTION

Hyperphenylalaninemias (HPAs) are heterogeneous group of autosomal recessive inborn errors of metabolism characterized by the inability to metabolize phenylalanine (Phe) due to enzyme defects of either phenylalanine hydroxylase (PAH) or its cofactors, resulting in possible consequent neurological damage.^1^, ^2^ The diagnosis is based on the finding of mutations in the *PAH* gene, with more than 1500 pathogenic variants reported to date (most frequently missense mutations), or other genes related to cofactor deficiencies (such as *QDPR*, *PTS*, *GCH1* and *PCBD1*).^13^ Variants in *DNAJC12* gene have also been recently described as causing possible hyperphenylalaninemia.^14^, ^15^, ^16^ Usually, patients with HPA are detected through newborn screening for phenylketonuria (PKU) and defined as affected by milder to more severe forms (PKU/HPA) depending on serum phenylalanine levels,^3^ which is due to a remarkable allelic variability accounting for different related patterns of phenylalanine hydroxylase residual enzymatic activity (EC 1.14.16.1).^4^, ^5^, ^6^ Prompt and correct identification/prediction of the hyperphenylalaninemia subtype represent the most important task on a clinical standpoint in order to set appropriate treatments, including in most cases a tailor‐made dietary intervention according to patients' needs, metabolic control and Phe tolerance.^7^, ^8^ *PAH* molecular pattern represent one of the mostly recognized predictive elements for possible metabolic outcomes. Furthermore, genotype/phenotype correlations have been reported with the expansion of a system of arbitrary values that, based on the assignment of an Allelic Phenotype Value (APV) to each *PAH* variant (GPV), seems to be capable of predicting the clinical phenotype with a validity index between 80% and 90%.^9^, ^10^, ^11^, ^12^

In this paper, we review the mutation spectrum of the hyperphenylalaninemic patients born since late ‘70 s in Lombardy, Italy, in follow‐up at the Clinical Department of Paediatrics, San Paolo Hospital, ASST Santi Paolo e Carlo, University of Milan, Italy, analysing allelic phenotype and genotype values (attributed on the basis of the APV/GPV system) to verify genotype–phenotype correlations and discussing potential clinical implications.

## 2 PATIENTS AND METHODS

After full anonymization, medical records of patients diagnosed with any form of hyperphenylalaninemia between 1955 and 2020 and in follow‐up at the Clinical Department of Paediatrics – San Paolo Hospital, ASST Santi Paolo e Carlo, University of Milan, Italy, were retrospectively reviewed and studied, including demographic details, indices of metabolic control and genetic analysis results.

Data regarding *PAH*, *QDPR*, *PTS*, *GCH1* and *PCBD1* analysis were collected considering genomic DNA previously extracted from whole blood samples with different techniques; *DNAJC12* analysis results were collected when available. Used techniques for genetic analysis included variably DGGE (Denaturing Gradient Gel Electrophoresis), direct sequencing, HRM (High Resolution Melt), Sanger sequencing, copy number deletions or duplications with Multiple Ligation Probe Amplification (MLPA, MRC Holland, Resnova) or Next‐Generation Sequencing (NGS), following technological advances over years (MiSeq platform ‐ Illumina and custom TrueSeq Custom Amplicon kit 2016–2018, Nextera Rapid Capture Custom enrichment chemistry starting from 2019–2020). Variants were described according to Human Genome Variation Society (HGVS) nomenclature guidelines (using Mutalyzer Name Checker tool) and classified as disease causing when described as pathogenic or likely pathogenic in Clinvar, PAHvdb, LOVD or HGMD databases. As PAHvdb is linked to the genotype–phenotype BIOPKU database (BIOPKU; http://www.biopku.org/biopku/search‐start.asp), this was also used for analyses, providing additional information including allelic phenotype values (APV). The accession number for *PAH* was RefSeq: ENSG00000171759; GeneBank: NM_000277.1. For newly identified variants, VarSite was used as data source^13^ with reference PAH protein sequence UNIPROT P00439. All novel variants were evaluated using ACMG classification and Varsome.^14^, ^15^, ^16^ New variants were considered ‘pathogenic’ or ‘likely pathogenic’ and reported after evaluating classification of functional Prediction tools (Mutation Taster, ACMG classification, Varsome, FATHMM, Varsite).

To predict phenotype in patients with PAH deficiency, results of Phe concentrations at the newborn screening were considered, including either dosages in dried blood spots (DBS) or venous puncture. For cases in which newborn screening details could not be retrieved due to incompleteness of documentation (including late diagnosed patients), available results of Phe concentrations prior to the start of any type of treatment were considered (either on DBS or by venous puncture).

For classification purposes, the BIOPKUdb proposed model (BIOPKU; http://www.biopku.org/biopku/search‐start.asp) was used, as follows: classicPKU (cPKU ≥ 1200 μmol/L), mildPKU (mPKU 600–1199 μmol/L) and mildHPA (mHPA < 599 μmol/L). Based on the European guidelines on phenylketonuria,^17^, ^18^ the distinction between forms ‘requiring treatment’ (that is pre‐treatment blood Phe levels ≥360 μmol/L) and ‘not requiring treatment’ (that is pre‐treatment blood Phe levels 120–360 μmol/L), was also applied to add more insights defining disease type and severity.^18^

The Allelic Phenotype Values (APV) system proposed by Garbade^5^, ^11^ was calculated and used to perform genotype/phenotype correlations thus, for each patient, APVs related to both pathogenic variants of *PAH*, as proposed in the BIOPKUdb and PAHvdb databases, were reported (APVs range between 0 and 10, identifying cPKU for APV 0–2.7, mPKU for APV 2.8–6.6 and mHPA for APV 6.7–10).^11^ Based on APVs, Genotypic Phenotype Values (GPVs) were also calculated and intended as the higher found APV (APVmax) between the two variants; this was considered applicable since the milder variant (that is with a higher APV) is contemplated as always dominant over the severe one.^11^, ^19^ Such type of evaluations were not performed for patients with no reported variants and/or with only a single variant reported (e.g., simple heterozygotes with a biochemical diagnosis of hyperphenylalaninemia), with the exception of patients in which the reported mutation was related to a GPV > 6.7 because, being the mildest one, the expected phenotype could not have been different since always dominant over the severe one.^11^, ^21^

Linear discriminant analyses (LDA) were thus computed to predict the clinical phenotype (cPKU, mPKU, and mHPA) starting from individuals' GPV: GPVs ≤ 2.7 were considered predictive for cPKU, GPVs 2.7–6.6 for mPKU and GPVs ≥ 6.7 for mHPA. Thus, a PKU‐variant (APV < 6.7), associated with an HPA‐variant (APV ≥ 6.7) should predict a clinical phenotype of mildHPA (GPV ≥ 6.7). To ensure GPV test's ability to generate reliable results, validity index was used for each individual to compare expected phenotype (based on GPV) with presenting phenotype; patients were thus considered ‘true positives’ when matching and ‘false positives’ when differing.

Possible effects of interallelic complementation, epigenetic factors and other possible elements which may influence phenotype,^20^, ^21^ were not considered in this study.

Statistical analysis was performed and graphed using R version 4.0.3, an open‐source software and flexible programming language used for the statistical data analysis as well as graphic creations.

All data in this study are reported as means ± SD or numbers (%) unless otherwise indicated. Phe values are reported as means ± SD, median, maximum and minimum for each group. The validity index was calculated as the ability of the GPV‐based test to generate results corresponding to the truth, thus as the percentage of patients with clinical phenotype matching the predicted phenotype, out of the total number of patients with a given predicted phenotype.

Informed written consent was provided from patients to have data from their medical records used in this research and data submissions and procedures were in accordance with the ethical standards while approved by local institutional review boards, where applicable.

## 3 RESULTS

### 3.1 Demographics

**FIGURE 1:**
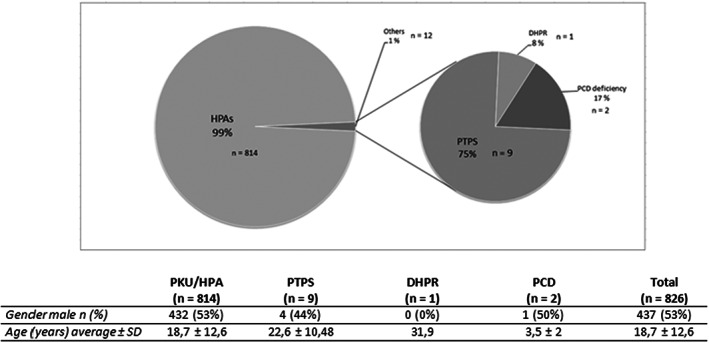
Distribution of patients based on type of hyperphenylalaninemia and demographics characteristics. Distribution of patients affected by any type of HPA and in follow up at the Clinical Department of Paediatrics, ASST Santi Paolo e Carlo, San Paolo Hospital, University of Milan, Italy, along with indications about gender and age distribution. Abbreviations: HPAs = hyperphenylalaninemias; PTPS = 6‐Pyruvoyl‐Tetrahydropterin Synthase Deficiency; DHPR = Dihydropteridine Reductase Deficiency; PCD = Pterin‐4 alpha‐carbinolamine dehydratase (PCD) deficiency.

Eight hundred and twenty‐six patients (*n* = 826) affected by different forms of hyperphenylalaninemia were actually in follow up at the Clinical Department of Paediatrics (ASST Santi Paolo e Carlo, San Paolo Hospital, University of Milan, Italy) at the initiation time of the study. Out of them, 814 were either PKU or HPA affected (99%), with genders nearly equally distributed and average age 18.75 ± 12.63 years, while the remaining part was constituted of nine patients affected by PTPS deficiency, one patient affected by DHPR deficiency, and two patients affected by PCD deficiency. Distribution of patients and demographics characteristics are summarized in Figure 1. Ethnicity was predominantly Caucasian, for both populations.

### 3.2 Molecular results

#### 3.2.1 *PAH* gene

Ninety three percent of patients (*n* = 758) in the evaluated cohort are affected either by phenylketonuria or hyperphenylalaninemia, for a total of 1519 *PAH* genotype records retrieved and 166 different genetic variants highlighted, distributed in all 13 exons and 3′UTR region. p.Ala403Val is the most frequently observed variant, present in 149 alleles (9.81%) out of the total genetic records collected. Other very frequently observed variants included: p.Arg261Gln in 126 alleles (8.29%); p.Val245Ala in 121 alleles (7.96%); IVS10‐11 g > a in 103 alleles (6.78%); p.Tyr414Cys and p.Leu48Ser in 84 alleles (5.53%) (TableS1). In a minor percentage of cases, variants in *PAH* seem absent (4%, *n* = 31) or appear to identify only heterozygosity (3%, *n* = 25), thus are not yet fully defined on a genetic basis in patients that still are clinically affected. Especially in such cases, MLPA copy number analysis technique resulted preferably used to elaborate on the case and in most cases concluding identifying deletions. As an example, the case of a particular deletion affecting exon 4 and initially escaping because under the primer and visible only in the forward sequence, thus not with the reverse primer. This was not in fact a true deletion, rather a mutation that fell into the splicing site just below the MLPA probe which, for this reason, could not be linked. After carrying out both PCR and sequencing according to Sanger of exon 4, the c.441 + 5G > T (IVS4 + 5 g > t) variant could be found (a well‐known and pathogenetic variant whose identification allowed to conclude the case as a double mutate with an already found mutation found in previous analysis).

Seven novel variants in seven different patients were identified (see Table 1), not found in gnomAD browser (minor allele frequency MAF =0): (1) c.272 T > C (p.Leu91Pro), (2) c.569 T > A (p.Val190Glu), (3) c.947 T > A (p.Glu316Val), (4) c.978G > C (p.Trp326Cys), (5) c.1075 T > C (p.Ser359Pro), (6) c.583A > G (p.Lys195Glu), except for (7) c.1013A > C (p.Asp338Ala) (MAF = 0.000003981). All variants are predicted as pathogenic/likely pathogenic in ACMG classification/Varsome. In all cases, the second associated variant was already known and described. Each of these patients was either diagnosed with HPA (*n* = 3) or PKU (*n* = 4) based on Phe levels.

**TABLE 1:** New *PAH* gene variants identified in our cohort of patients. Specifications about newly identified variants (on both alleles) and related effects in patients affected by HPAs in current follow‐up at the Clinical Department of Paediatrics, San Paolo Hospital, ASST Santi Paolo e Carlo, University of Milan, Italy.

	Allele 1 variant	Allele 1 effect	Allele 2 variant (Effect)	Predicted effect (ACMG classification/varsome)
***1***	c.272 T > C	**p.Leu91Pro**	c.688G > A (p.Val230Ile)	pathogenic
***2***	c.569 T > A	**p.Val190Glu**	nt45delCT (p.Ser16Ter)	pathogenic
***3***	c.947 T > A	**p.Glu316Val**	c.782G > A (p.Arg261Gln)	pathogenic
***4***	c.978G > C	**p.Trp326Cys**	c.1222C > T (p.Arg408Trp)	likely pathogenic
***5***	c.1075 T > C	**p.Ser359Pro**	c.1222C > T (p.Arg408Trp)	likely pathogenic
***6***	c.583A > G	**p.Lys195Glu**	c.1157A > G (p.Tyr386Cys)	pathogenic
***7***	c.1013A > C	**p.Asp338Ala**	c.842C > T (p.Pro281Leu)	likely pathogenic

The c.272 T > C (p.Leu91Pro) variant was found in one patient in association with a second variant (p.Val230Ile) with an APV = 10, thus cannot predict alone the expected phenotype. This patient has pre‐treatment Phe levels only mildly elevated (199 μmol/L) and is currently not requiring dietary treatment. This variant is predicted as ‘pathogenic’ in ACMG classification/Varsome.

The c.569 T > A (p.Val190Glu) variant was found in one patient in association with a second variant (p.Ser16Ter). This patient has pre‐treatment Phe levels considerably elevated (2578 μmol/L) and is currently on dietary regimen with a Phe tolerance of 283 mg Phe/day up to date (3 years of age). This new variant is predicted as ‘pathogenic’ in ACMG classification/Varsome. As the second variant in this case has an APV = 0, p.Val190Glu may be suggestive for cPKU.

The c.947 T > A (p.Glu316Val) variant was found in one patient in association with a second variant c.782G > A (p.Arg261Gln). In this patient, pre‐treatment Phe level were only slightly elevated (164 μmol/L) and a dietary intervention has never been required up to date. This variant is predicted as ‘pathogenic’ in ACMG classification/Varsome. Since the second variant has an APV = 1.6, p.Glu316Val may be predictive for mildHPA.

The c.978G > C (p.Trp326Cys) variant was found in association with a second variant c.1222C > T (p.Arg408Trp) in one patient that showed high pre‐treatment Phe levels (942 μmol/L) and is currently undergoing dietary treatment, with a Phe tolerance of 340 mg Phe/day up to date (3 years of age). This variant is predicted as ‘likely pathogenic’ in ACMG classification/Varsome. Since the second variant in this case is linked to an APV = 0, it can be expected that this new variant may be linked to mild PKU.

The c.1075 T > C (p.Ser359Pro) variant was found in a patient that demonstrated very high pre‐treatment Phe levels (2488 μmol/L) and is currently on dietary treatment (Phe tolerance = 250 mg Phe/day at 3 years of age). This variant is predicted as ‘likely pathogenic’ in ACMG classification/Varsome. In this patient, the second found variant is c.1222C > T (p.Arg408Trp), which is linked to an APV = 0 thus this new variant may be indicative of cPKU.

The c.583A > G (p.Lys195Glu) variant was found in one patient with high pre‐treatment Phe levels (596 μmol/L) in association with a second variant c.1157A > G (p.Tyr386Cys). This patient is currently on dietary treatment with a Phe tolerance of 550 mg Phe/day at 3 years of age. p.Lys195Glu is predicted as ‘pathogenic’ in ACMG classification/Varsome. Since the second variant is linked to an APV = 0, it is possible that this new variant is predictive of mildHPA.

The c.1013A > C (p.Asp338Ala) variant was found in one patient with only slightly elevated pre‐treatment Phe levels (143 μmol/L) and without the need for any type of medical intervention. This new variant is predicted as ‘pathogenic’ in ACMG classification/Varsome. The second variant in this case is c.842C > T (p.Pro281Leu), which is linked to an APV = 0 thus this new variant may be indicative for mildHPA.

All novel variants with predicted effects explanation are presented in Table 1. For all these novel variants, as they were found in compound heterozygosis with other common variants reported as pathogenic in ClinVar and related to clinically affected patients, pathogenicity is strongly suggested.

#### 3.2.2 *PAH* gene associated phenotypes

**FIGURE 2:**
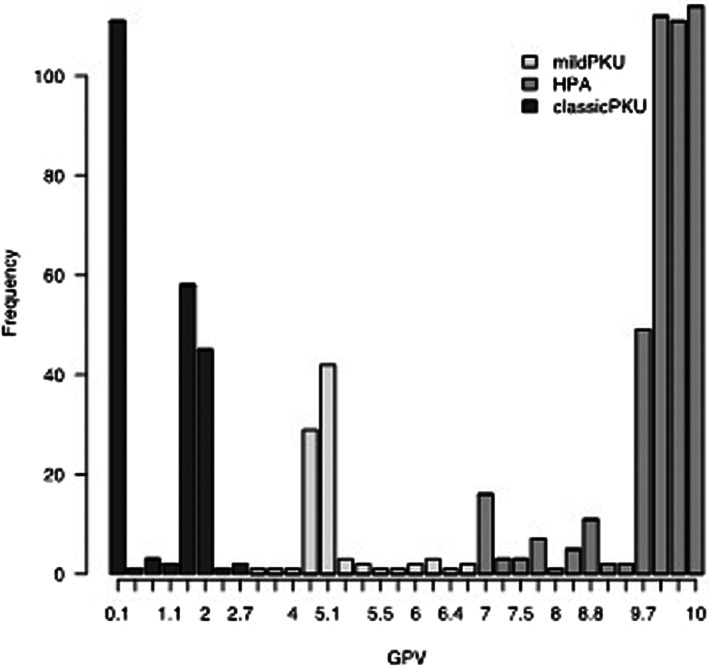
Distribution of according to GPV. Distribution of patients is represented with data expressed as absolute values, based on identified GPV value and classified in different forms of hyperphenylalaninemia (mild PKU, hyperphenylalaninemia = HPA and classic PKU). Calculation of expected phenotype was obtained from the site www.biopku.org.

In this study, APV and GPV could be calculated for a total of 748 of patients (92% of sample size) differentiating our cohort in classicPKU (cPKU, *n* = 223, for GPV ≤ 2.7), mildPKU (mPKU, *n* = 89, for GPV 2.7 – 6.7) and mildHPA (mHPA, *n* = 436, for GPV ≥ 6.7) based on expected phenotype, as represented in Figure 2.

**FIGURE 3:**
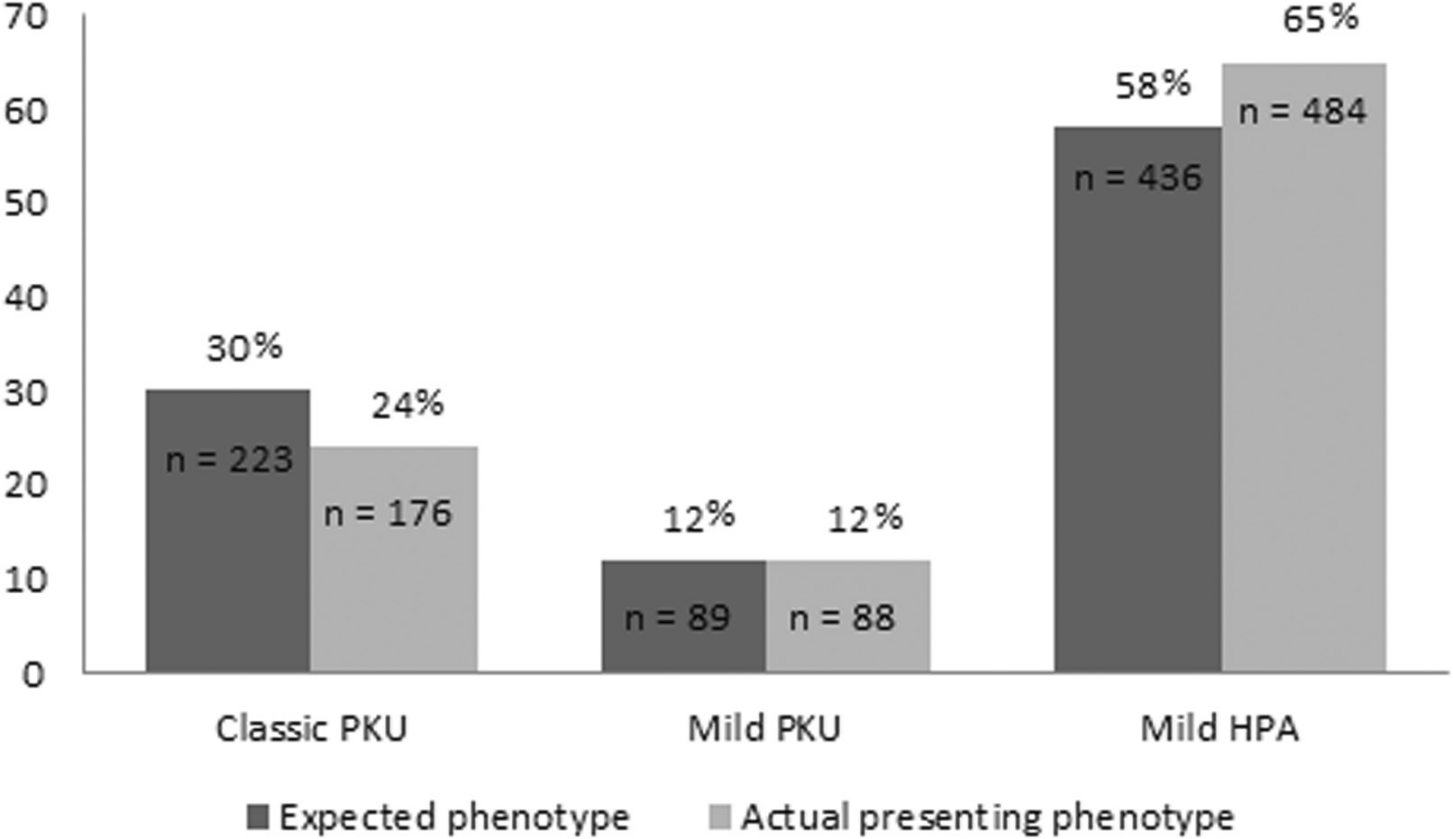
Discrepancies in ‘expected’ vs. ‘presenting’ phenotype based on type of HPAs. Evidence of relevant discrepancies between type of HPA prediction and reality: on the left, the distribution of the expected phenotypes according to the GPV; on the right, the distribution of actual presenting phenotype based on pre‐treatment Phe levels, divided according to Blau's classification.

Predictions were reliable with the presenting phenotype only in 85% of cases (*n* = 638) according to Blau et al.'s classification,^10^ with an identified relevant discrepancy between expected results and actual ones (specific data about distribution of PKU/HPA patients is given in Figure 3).

Specifically:

1. *GPV ≤ 2.7 (expected phenotype = cPKU)*. In this category, phenotype could be reliably predicted in 76% of cases (*n* = 169) infact presenting with a cPKU form (pre‐treatment blood Phe > 1200 μmol/L). In the remaining 24% of cases (*n* = 54), GPV was not reliable: 17% of cases (*n* = 38) presented with a mildPKU form (pre‐treatment blood Phe 600–1200 μmol/L) and 7% (*n* = 16) with a mildHPA one (pre‐treatment blood Phe < 600 μmol/L). For those presenting with a mildPKU form, a dietary treatment was needed, as expected based on pre‐treatment levels (mean blood‐Phe levels 745.45 ± 112.08). Besides that, this was not as restricted as expected for a classic form in terms of tolerance. Particularly, for one patient, found homozygous for p.Ala104Asp, a dietary treatment was needed but related dietary tolerance was 650 mg Phe/day at 6 years, with corresponding mean blood Phe levels demonstrating good metabolic control (<360 μmol/L). In 2 other patients, both homozygous for p.Leu48Ser, mean blood Phe levels were 854.97 ± 126.13 μmol/L and, for one of them, pharmacological treatment with sapropterin dihydrochloride could be unexpectedly applied resulting in optimal metabolic control (<360 μmol/L) and a dietary tolerance of 900 mg Phe/day to date. With regards to patients presenting instead with a mild HPA form, 11 patients had pre‐treatment Phe levels of 360–600 μmol/L and 5 patients <360 μmol/L (mean 167.92 μmol/L, min 140.20 μmol/L max 215.50 μmol/L); of the latter, no patients required dietary intervention at least up to date. Following variants were identified in these patients: compound heterozygosity (p.Arg261Gln;p.Tyr386Cys) for 2 patients, compound heterozygosity (p.Arg408Trp; IVS10‐11 g > a) for 1 patient and homozygosity (p.Leu48Ser) for the remaining 2 patients. All these variants are reported in literature as normally linked to a cPKU phenotype. p.Leu48Ser variant was frequently observed in cases with phenotypes differing from those expected; in particular, this occurred in 40% of the cases of homozygosity for p.Leu48Ser, suggesting uncertainty for its definition as ‘classic’ or else a possible variable expressiveness.

2. *GPV 2.7–6.7 (expected phenotype = mild PKU)*. In this category, phenotype could be reliably predicted only in a minor percentage of patients (48%, *n* = 43). In the remaining 52% of patients (*n* = 46), 7% (*n* = 6) unexpectedly presented with a cPKU form and 45% (*n* = 40) with a mild HPA. Focusing on patients that presented with a mildHPA form, pre‐treatment blood Phe levels were 360–600 μmol/L in 31 patients, while <360 μmol/L in the remaining nine patients. Five of the latter never required any kind of intervention (mean blood Phe levels 212.3 μmol/L; min 144.50 μmol/L, max 262.00 μmol/L) and the following genetic variants were found: patient (1) p.Pro119Ser in homozygosity, patient (2) p.Ala395Gly in homozygosity, patient (3) compound heterozygosity for p.Pro281Leu and p.Lys396Arg, patient (4) compound heterozygosity for p.Arg158Trp and Ala309Val, patient (5) compound heterozygosity for p.Tyr414Cys and p.Leu41Phe. With the aim to best address genetic insights, we deepened the analysis with regards to variants found in homozygosity, for whom a clear connection with presenting phenotype was more applicable. p.Pro119Ser and p.Ala395Gly, when in homozygosity, presented with pre‐treatment blood‐Phe levels of 144 and 192 μmol/L, respectively, still remaining stable without need of dietary intervention.

The p.Tyr414Cys variant was also frequently observed in cases with phenotypes differing from those expected (mildHPA instead of mild PKU); in particular, this occurred in 64% of the cases of homozygosity for p.Tyr414Cys, suggesting uncertainty for its definition as ‘mild PKU’ or else a possible variable expressiveness.

With regards to patients expected to carry a mildPKU form and instead presenting with a cPKU one, p.Leu333Phe was found in 2 siblings, both homozygous, with pre‐treatment blood‐Phe levels of 1612.00 ± 520 μmol/L and requiring dietary intervention.

3. *GPV ≥ 6.7 (expected phenotype = mild HPA)*. In this category, phenotype could be reliably predicted in 98% of cases; remaining part was 8 patients with a mild PKU phenotype and 2 patients with cPKU. p.Glu390Gly was observed in all cases presenting with a mild PKU phenotype, variably associated with other variants all reported as linked to classic forms. This observation suggest uncertainty for the definition of p.Glu390Gly as a ‘mild HPA’ variant or else a possible variable expressiveness.

With regards to the two latter patients reported with a unexpected cPKU phenotype, these were both females with compound heterozygosity (patient 1: p.Arg158Gln and p.Ala300Ser; patient 2: p.Arg261Gln and p.Ala300Ser); pre‐treatment blood Phe levels were 1368 and 1400 μmol/L, respectively. The first subject is confirming her clinical phenotype overtime and is on dietary treatment still. Her dietary Phe tolerance was 270 mg/day at 1 year of life and reached 350 mg Phe/day at 6 years of life; as expected based on blood pre‐treatment Phe levels and BH4 loading test performed, no responsiveness to sapropterin could be demonstrated though both variants are currently described as BH4‐responsive in literature.^22^ The second subject, on the contrary, despite high pre‐treatment blood Phe levels but carrying possibly responsive variants, was confirmed responsive at the BH4 loading test with a much higher Phe tolerance than expected, increasing overtime (400 mg Phe/day at 1 year of life reaching 1200 mg Phe/day at 3 years old, when put on sapropterin); still, a liberalization of the diet has not been gained.

Despite a highly positive predictive value for GPV ≥ 6.7, it has to be considered that this specific category includes, based on Blau's classification,^10^ both patients who need some type of treatment and patients who do not, and that even if high GPV seem to be very predictive for a mild HPA form, it cannot discriminate alone who is going to need treatment and who's not. As this can result in possible relevant difficulties managing the patient at the newborn screening, we felt that this population could be better assessed in defining prediction discrepancies based on a different classification, thus dividing patients into those ‘not requiring treatment’ (90%, *n* = 394) and those ‘requiring treatment’ (10%, *n* = 42), in accordance with European guidelines.^18^ When divided as illustrated, it became even more evident how much GPV is reliable defining mild forms but that, in numerical terms, this category of patients has decidedly higher possibilities of not needing diet therapy than needing it. We then tried to identify possible GPV cut offs that could help differentiate among the two differently identified subgroups. For this purpose, we tried to apply the use of a ROC curve for the identification of a GPV cut‐off value capable of discriminating between patients requiring treatment and not but failed. It was in fact not possible to identify this specific threshold value, as the only effective identified cut off (calculated on a Youden's Index basis^23^) was associated to a GPV < 9, thus not useful on a clinical standpoint (the aim was to help discriminate among patient with GPV ≥ 6.7 in a more strict way). Even when we tried to construct GPV percentiles based on phenotype (dividing into the three different identified categories), it was not possible to delimit the various categories with a 95% confidence interval. This made it impossible to identify with a certainty range the delimitation, therefore the prediction of the clinical phenotype only according to the GPV.

Aiming to add more insights on this specific subgroup of patients, we can state that mean Phe concentration among patients with GPV ≥ 6.7 was 241.46 ± 136.25 mmol/L (min 102.8; max 1014.0). Dividing patients based on Phe plasma levels (cut‐off 360 mmol/L) we could find that for patients ‘requiring treatment’ (10%, *n* = 42), mean Phe levels were 513.85 ± 145.23 mmol/L and predominant genotypes were: Y414C (allele frequency 49.12%), L48S (allele frequency 38.6%), R261Q (allele frequency 10.53%), R408W (allele frequency 7.02%), R158Q (allele frequency 5.26%) and IVS12 + 1 g‐ > a (allele frequency 5.26%). With regards to patients ‘not requiring treatment’ (90%, *n* = 394), mean Phe levels were 195.80 ± 60.20 mmol/L and predominant genotypes were: A403V (allele frequency 36.47%), V245A (allele frequency 31.17%), A300S (allele frequency 13.52%), R261Q (allele frequency 10%) and Y414C (allele frequency 7.12%).

### 3.3 Other molecular results

#### 3.3.1 >BH4 deficiencies associated genes

Out of the total of patients reviewed over a time frame of 65 years, 12 are carrying mutations in genes involved in some type of BH4 deficiency (1% of patients, which is in line with reported literature where tetrahydrobiopterin deficiencies account for 1%–3% of all cases of elevated phenylalanine levels). In these cases, a different diagnosis was expected based on previously collected pterins results. Patients were diagnosed as follows: nine patients affected by PTPS deficiency (and identified *PTS* variants), two patients affected by Pterin‐four alpha‐carbinolamine dehydratase deficiency (and identified *PCBD1* variants) and one patient affected by DHPR deficiency (and identified *QDPR* variants). There were no patients tested for GCH1 gene mutations, as none showed compatible alterations on a pterins level. Variants identified in our cohort of BH4 deficient patients, along with details regarding values presented at screening (both in terms of phenylalanine and pterins), are indicated in Table 2 and have already been described in current literature.^24^, ^25^

**TABLE 2:** Variants identified in patients with BH4 deficiencies in our cohort. Variants identified in patients affected by BH4 deficiency in our cohort, including patients affected by PCD, DHPR or PTPS deficiencies.

Patient #	Enzyme defect (gene)	Allele 1 variant (Effect)	Allele 2 variant (Effect)
*1*	DHPR (*QDPR*)	c. 529A > G (p. Tyr150Cys)	c. 741C > T (p.Arg221Ter)
*2*	PTPS (*PTS*)	c.393del (p.Lys131fs*64)	g.3760_3816del (IVS2‐762‐718del)
*3*	PTPS (*PTS*)	c.393del (p.Lys131fs*64)	g.3760_3816del (IVS2‐762‐718del)
*4*	PTPS (*PTS*)	c.260C > T (p.Pro87Leu)	c.308 T > C (p.Val103Ala)
*5*	PTPS (*PTS*)	c.260C > T (p.Pro87Leu)	c.308 T > C (p.Val103Ala)
*6*	PTPS (*PTS*)	c.317C > T (p.Thr106Met)	c.308 T > C (p.Val103Ala)
*7*	PTPS (*PTS*)	c.240G > T (p.Met80Ile)	c.164‐37dup (IVS2‐37dup)
*8*	PTPS (*PTS*)	c.308 T > C (p.Val103Ala)	c.379C > G (p.Leu127Val)
*9*	PTPS (*PTS*)	c.370G > T (p.Val124Leu)	c.164‐1G > C (IVS2‐1 g > c)
*10*	PTPS (*PTS*)	c.146A > G (p.His49Arg)	c.244‐8G > C (IVS4‐8 g > c)
*11*	PCD (*PCBD1*)	c.172G > A (p.Glu58Lys)	c.179 T > C (p.Leu60Pro)
*12*	PCD (*PCBD1*)	c.172G > A (p.Glu58Lys)	c.179 T > C (p.Leu60Pro)

### 3.4 >DNAJC12 gene

*DNAJC12* analysis resulted as performed for those cases were copy number deletion/duplication analysis nor NGS sequencing could lead to conclusive results despite evidence of higher‐than‐normal Phe levels. None of our patients re‐tested to date did highlight any variant in *DNAJC12*.

## 4 DISCUSSION

This study aims to investigate the genotypic characteristics of the population of patients affected by hyperphenylalaninemia in follow‐up at the reference clinical centre of the Lombardy region, Italy. This cohort of patients appears to be one of the numerically largest in our nation as well as in Europe, therefore it constitutes a representative sample of the PKU affected population worldwide. Eight hundred and twenty‐six patients affected by different forms of hyperphenylalaninemia are in follow up at the Clinical Department of Paediatrics (ASST Santi Paolo e Carlo, San Paolo Hospital, University of Milan, Italy) and included in this study. Eight hundred fourteen are either PKU or HPA affected (49% of patients currently on dietary treatment, thus PKU), while the remaining part includes patients affected by various types of BH4 deficiency (nine patients affected by PTPS deficiency, one patient affected by DHPR deficiency and two patients affected by PCD deficiency). With regards to *PAH* genotype, 166 different genetic variants have been identified in this study. p.Ala403Val is the most frequent, found in 149 alleles (9.81%) out of the total of genetic records collected; this result is in line with what already found in current literature with regards to the Italian population.^25^, ^26^ Other very frequently observed variants includes: p.Arg261Gln in 126 alleles (8.29%); p.Val245Ala in 121 alleles (7.96%); IVS10‐11 g > a in 103 alleles (6.78%); p.Tyr414Cys and p.Leu48Ser in 84 alleles (5.53%). These latter results are in contrast with actual known genotype distribution in European PKU patients, among whom the most common variant, as reported in a recent study by Hillert et al.,^5^ is p.Arg408Trp, with an allelic frequency of about 64%, followed by c.106611G > A and p.Arg261Gln.

Patients are compound heterozygotes in 82% of cases, homozygotes in 13% and simple heterozygotes in the remaining 5% of cases. Seven novel variants have been also identified in our cohort. The identification of these new variants is relevant for the scientific field, as they can be included in current used database and improve and increase knowledge about patients affected by hyperphenylalaninemia.

In our study, we tried to predict phenotype based on the Allelic Phenotype Values and Genotypic Phenotype Values (APVs‐GPVs system).^10^, ^11^ According to Blau et al.'s classification, actual cases were matching with related prediction in most cases, but not all of them (85% of cases, *n* = 638), identifying a relevant discrepancy between expected results and presenting ones. Aiming to identify possible explanations, we proceeded evaluating patients after dividing them in different cohorts based on GPV, further clarifying that the APVs‐GPVs system may be helpful for clinical purposes but it may not be as representative as expected to when taken alone. We could actually identify, for example, patients with very low GPVs (<2.7) expected to be very severe clinically, instead presenting with low blood‐Phe levels that did not require dietary intervention.

There were some variants more associated with unexpected results. This included p.Leu48Ser. If imagined as in a functional hemizygote, this variant would be assigned to a phenotype of classic phenylketonuria (GPV < 2.7) and would seem to have a residual enzymatic activity of almost zero if associated with a ‘null allele’. Besides that, among our population this variant was found in two patients with mild PKU and in two patients with mild HPA, probably indicating a higher residual enzymatic activity than expected. Again, other variants were frequently observed in cases with phenotypes other than those expected. This is the case of the p.Tyr414Cys variant, that presented with a mildHPA phenotype instead of mild PKU, and the p.Glu390Gly variant, that emerged variably associated with other mutations defined as mild HPA based on GPV but instead presenting with a mild PKU form based on pre‐treatment Phe levels.

Such observations suggest uncertainty for a strict definition of these specific variants as done to date and suggest on the counterpart variable expressiveness due to possible intrinsic characteristics.

We also tried to identify possible GPV cut‐off values capable of ameliorate discrimination abilities in order to better predict prognosis, but this was not possible as the only found threshold was associated to a GPV not useful on a clinical standpoint. These limits said, we can only speculate that, considering GPVs, patients with pre‐treatment blood Phe levels  < 360 μmol/L had higher GPV value (mean 9.7, range 7–10) than patients with pre‐treatment blood Phe levels ≥360 μmol/L (mean 8.9, range 7–10).

We can add that when presenting phenotypes are differing from expected ones, this can also be due to the possibility that some mutations may be in deep intronic regions or in sequences that are difficult to study with the actual available means, thus the direct study of the RNA by looking for alternative splicing or considering interactions with a specific noncoding RNA should be considered; alternatively, the ‘switching off’ of one of the two alleles by transcriptional regulatory mechanisms may be supposed. This may be considered also for such patients where only one variant could be identified and yet are presenting with severe phenotypes.

Furthermore, positive or negative interallelic complementation or epigenetic factors should be taken into account, such as non‐genetic factors that affects phenotypic expression that may also have come into play, although, to date, they are still yet to be fully known. This includes considerations about recent works that stress about mechanistic heterogeneity of *PAH* dysfunction as a key factor when dealing with phenotype expression. For example, the sequence/structure predictive methods rely too heavily on the one sequence‐one structure‐one function assumption that lies at the basis for much bioinformatics analysis, explaining the ‘why’ of the frequent failure of current predictions.^27^ Furthermore, PAH structure is multimeric, usually a tetramer, with different mole fractions of the different *PAH* variants and even possible regulation processes for protein degradation,^28^, ^29^, ^30^ thus some predictive methods may ignore such tertiary and quaternary possible interactions.^31^, ^32^, ^33^ Mechanism is also linked to PAH allostery, which involves high levels of Phe causing an activation associated with major conformational changes, thus allosteric failure may explain why PKU‐associated variations can occur throughout the *PAH* sequence.^34^ With regards to the latter, our paper may already even more confirm this assumption, as it identified p.Leu48Ser (based in the region of the protein that repositions and forms the binding site for allosteric Phe) and p.Tyr414Cys (whose environment is predicted to change in the transition from the auto‐inhibited resting state to the activated state) as having clinical outcomes poorly predictable, which can be thus related to the structural basis of PAH allosteric activation.

Taken all together, these limitations of yet to be fully known underlying mechanisms should be accounted when considering possible genotype–phenotype correlations.

Among our population there are still some patients currently on dietary treatment but lacking to identify any kind of mutation within analysed genes, including *DNAJC12*. These patients are expected to be carrying causal variants not identified with the current sequencing technologies or, alternatively, to be carrying mutations in other genes yet to be identified.

## 5 CONCLUSIONS

Hyperphenylalaninemia is a very complex family of metabolic disorders, characterized by a multitude of genetic variations. This paper summarizes past 65 years genetic analysis of 826 patients with HPA/PKU from a single metabolic centre in Lombardy, Italy, identifying 7 new variants in the *PAH* gene and comparing metabolic phenotype with the APV genotype‐prediction. Many differences and discrepancies were pointed out in different categories, addressing the value and limitations of a variety of predictive tools that are currently used to direct clinicians in the management of such patients.

GPV, although capable of defining predicted phenotype with reasonable appropriateness based on its numerical value, seems not sufficient to characterize the patient with certainty into a defined category and, taken alone, it may not be as representative as expected to. Even within the same GPV category in fact, there are patients who differ significantly one another regardless of the same calculated GPV. This could be connected to the presence of some specific mutations, recurring with relation to the category, which could result in a decisive alteration of the phenotype, thus in need of much more clinical attention when occurring. Other variants in the same patient, not identifiable with current available systems, should also be accounted as possible responsible for mismatching.

Obviously, the deepening of knowledge in this sense could result in further improvements as well as making significant changes in the approach to the patient to better define his subsequent treatment needs.

Based on our results, we can conclude that no current predictive method is appropriate in more than 50% of the patients, thus there is still a growing need to identify other elements that could help redefine and better characterize such cases for conclusive diagnostic/therapeutic approaches.

Further studies are needed to improve and collect increasing evidence regarding this topic.

## AUTHOR CONTRIBUTIONS

Conceptualization: Valentina Rovelli, Davide Bassi and Juri Zuvadelli; Data curation: Juri Zuvadelli, Alice Re Dionigi, Daniela Graziani, Olivia Turri, Luisella Alberti and Valentina Rovelli; Formal analysis: Vittoria Ercoli, Daniela Graziani and Juri Zuvadelli; Investigation: Valentina Rovelli, Juri Zuvadelli, Daniela Graziani and Vittoria Ercoli; Methodology: Vittoria Ercoli and Daniela Graziani; Project administration: Valentina Rovelli; Supervision: Valentina Rovelli and Giuseppe Banderali; Validation: Valentina Rovelli, Vittoria Ercoli and Juri Zuvadelli; Visualization: Valentina Rovelli, Vittoria Ercoli and Juri Zuvadelli; Writing – original draft: Valentina Rovelli, Davide Bassi, Daniela Graziani, Olivia Turri; Writing – review & editing: Sabrina Paci, Elisabetta Salvatici, Raed Selmi, Graziella Cefalo, Alice Re Dionigi, Valentina Rovelli and Giuseppe Banderali.

## FUNDING INFORMATION

This research received no external funding.

## CONFLICT OF INTEREST

The authors declare no conflict of interest.

## ETHICAL APROVAL

The study was conducted according to the guidelines of the Declaration of Helsinki and with D.L. 196/2003 and the guidelines for the processing of personal data of our clinical institution.

## Supporting information


Table S1

## Data Availability

The datasets generated during and/or analysed during the current study are available from the corresponding author on reasonable request.
