# Supplementary material for: Hyperphenylalaninemias genotyping: Results of over 60 years of history in Lombardy, Italy
Source: Endocrinol Diabetes Metab. 2022 Dec 19;6(2):e396. doi: 10.1002/edm2.396 (PMC10000623; doi:10.1002/edm2.396)
Supplement: Supplementary file 1 — Table S1 [file EDM2-6-e396-s001.docx]

# SUPPLEMENTARY MATERIAL.

Table 1 (S1). At a glance overview of most frequently observed variants among our PKU patients, considering only variants with a PAH gene allele frequency > 10.

| **Variants** | **Absolute frequency** | **% Frequency** | **Simple heterozygosity** | **Compound heterozygosity** | **Homozygosity**  **(n)** |
| --- | --- | --- | --- | --- | --- |
| A403V | 149 | 89,8 | 3 | 112 | 17 |
| R261Q | 126 | 75,9 | 7 | 103 | 8 |
| V245A | 121 | 72,9 | 1 | 108 | 6 |
| IVS10-11g->a | 103 | 62 | 3 | 78 | 11 |
| Y414C | 84 | 50,6 | 2 | 60 | 11 |
| L48S | 84 | 50,6 | 3 | 61 | 10 |
| P281L | 72 | 43,4 | 3 | 61 | 4 |
| R408W | 64 | 38,6 | 1 | 39 | 12 |
| A300S | 61 | 36,7 | 2 | 59 |  |
| R158Q | 56 | 33,7 | 2 | 50 | 2 |
| V230I | 35 | 21,1 |  | 33 | 1 |
| T380M | 26 | 15,7 |  | 24 | 1 |
| E390G | 25 | 15,1 | 1 | 22 | 1 |
| S16X | 20 | 12 |  | 20 |  |
| R252W | 18 | 10,8 |  | 18 |  |
| IVS12+1g->a | 16 | 9,6 | 1 | 15 |  |
| D415N | 16 | 9,6 | 2 | 12 | 1 |
| R261X | 15 | 9 |  | 15 |  |
| E178G | 13 | 7,8 |  | 13 |  |
| S349P | 11 | 6,6 |  | 11 |  |
| R53H | 11 | 6,6 | 1 | 10 |  |
